# A chemiluminescent lantern: a coordination cage catalysed oxidation of luminol followed by chemiluminescence resonance energy-transfer†

**DOI:** 10.1039/d3dt00689a

**Published:** 2023-03-08

**Authors:** Atena B. Solea, Michael D. Ward

**Affiliations:** a Department of Chemistry, University of Warwick Coventry CV4 7AL UK m.d.ward@warwick.ac.uk

## Abstract

A molecule of luminol bound as guest inside a Co_8_ coordination cage host undergoes oxidation by H_2_O_2_ to generate chemiluminescence by a process in which the Co(ii) ions in the cage superstructure activate the H_2_O_2_: accordingly the cage not only co-locates the reactants but also acts as a redox partner in the catalysis. The luminescence from oxidation of the cavity-bound luminol can transfer its excitation energy to surface-bound fluorescein molecules in an unusual example of Chemiluminescence Resonance Energy Transfer (CRET).

## Abstract 2

Catalysed oxidation of luminol by H_2_O_2_ inside the cavity of a coordination cage is facilitated by a Co(ii)/Co(iii) redox couple from the cage superstructure.

## Graphical abstract

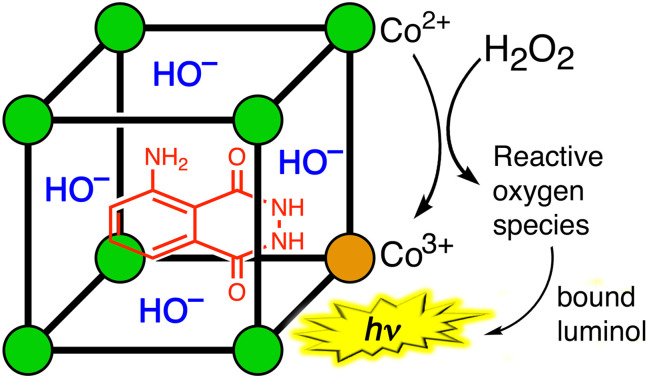

## Introduction

Hollow self-assembled metal/ligand coordination cages^1^ continue to provide a highly fertile basis for the study of host–guest chemistry and the development of functions associated with guest encapsulation, particularly catalysis, which is often based on the bound guest being in a substantially different environment from the bulk solution phase.^2^ If the components of the cage – the metal ions and bridging ligands – incorporate desirable photophysical or redox characteristics, then such cages can act as more than just molecular containers and can take an active role in photochemical reactions of bound guests.^3^ Coordination cages are particularly appropriate for such studies as a large number of chromophoric and/or redox active component parts may be combined in a compact assembly in close proximity to a bound guest, which means that a guest may be surrounded by a local concentration of such reaction partners which would be impossible to attain under normal solution conditions. This is the basis of some recent examples of photo-redox catalysis using coordination cage hosts.^4^

Expanding further the ways in which potential reaction partners can be combined in a single self-assembled array, we have shown recently that binding sites associated with the *external* surface of our cubic M_8_L_12_ coordination cage host^5–10^ can bind aromatic anions such as fluorescein^6^ or phenolates^7^ strongly in water, a process which is quite distinct from (and orthogonal to) binding of neutral hydrophobic guests in the cage central cavity.^8^ This means that we can simply form a supramolecular assembly which contains four different types of component in well-defined environments: eight metal ions and twelve ligands in the cage superstructure; a cavity-bound guest; and multiple (potentially, up to six)^6^ surface-bound anionic guests. Each of these can be chosen to have desirable redox or photophysical properties. Vertex metal ions can be purely structural (Zn^2+^, Cd^2+^), or redox-active (Ru^2+^),^5^ or can provide the basis for long-lived excited states (Os^2+^);^9^ the bridging ligands incorporate naphthyl fluorophores;^5^ cavity-bound guests can be energy- or electron-acceptors;^9,10^ and the external surface-bound anions can likewise be one of a wide range of organic fluorophores.^6^ With four types of tunable component integrated into a spatially well-defined supramolecular array, the scope to integrate their individual properties to develop sophisticated forms of reactivity are substantial.

We report here a significant step forwards in our development of the catalytic properties of cage-based supramolecular assemblies, in the form of a chemical oxidation reaction of a bound guest (luminol) by H_2_O_2_ which is signalled by appearance of chemiluminescence (CL) and requires redox participation of the cage metal ions: further, we demonstrate an unusual example of CL-based resonance energy-transfer (CRET) between donor and acceptor components that are held in close proximity by their differing interactions with the cage.

## Results and discussion

**Fig. 1:**
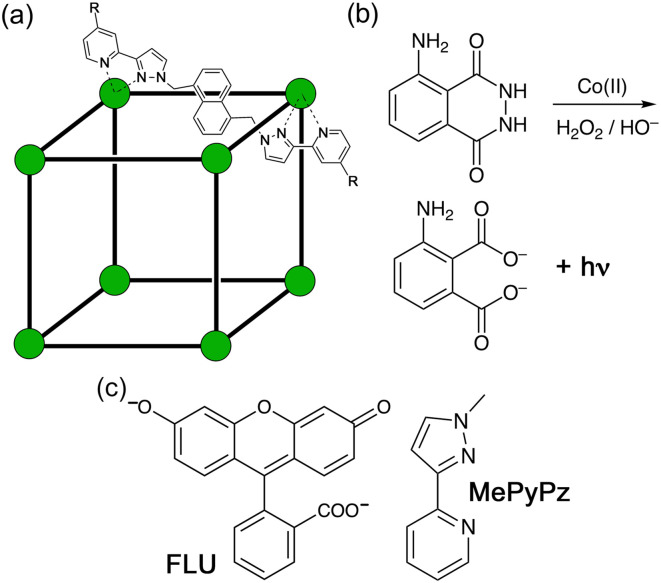
(a) Sketch of the [M_8_L_12_]Cl_16_ cubic coordination cage M·H^w^ (M = Co for the catalysed reaction, or Zn for the control experiment, see main text) and R = CH_2_OH; (b) the chemiluminescence-generating reaction of luminol with H_2_O_2_; (c) structures of FLU and MePyPz.

Luminol (Fig. 1) is fluorescent, and additionally exhibits blue chemiluminescence (CL) when oxidised, which persists for many minutes and is used as an analytical tool in forensic investigations.^11^ H_2_O_2_ is commonly used as the oxidant, although it does not immediately react with luminol: the H_2_O_2_ requires activating by a redox process with a catalyst,^12^ which includes any of a wide range of metal ions, to generate reactive oxygen species (ROS) which then oxidise luminol.^11^ This is the basis of its forensic use: a mixture of luminol and H_2_O_2_ is sprayed to test for traces of blood, with the iron ions in the haemoglobin activating H_2_O_2_ and triggering the CL response.^11*a*^ Luminol is of a size commonly associated with guest binding in our M_8_L_12_ cage host (Fig. 1), with a molecular volume of 137 Å^2^ (*cf.* cavity volume, 409 Å^3^).

**Fig. 2:**
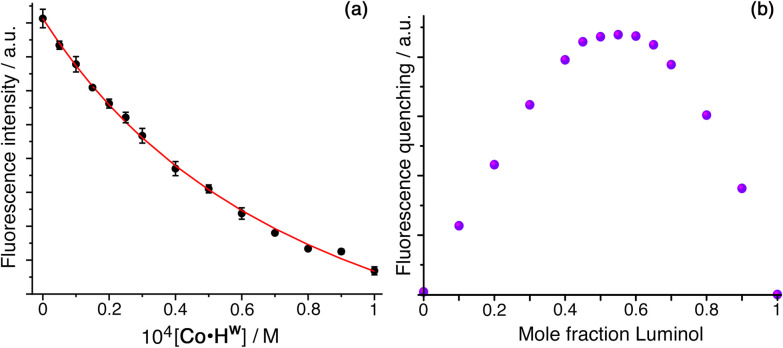
(a) Luminescence titration involving addition of increasing amounts of Co·H^w^ to luminol (10 μM) in water, showing quenching of luminol fluorescence as it is taken up into the cage cavity (*λ*_exc_ = 350 nm); (b) a Job plot showing the degree of fluorescence quenching at different Co·H^w^/luminol mole fractions, confirming 1 : 1 binding.

Addition of portions of **Co·H**^**w**^ (rendered water-soluble by attachment of hydroxy groups to the exterior surface) to a solution of luminol in water resulted in progressive uptake of luminol and quenching of its native photo-luminescence. Fitting the data (Fig. 2a and S1†) to a 1 : 1 binding isotherm gave *K* = 1.26(6) × 10^4^ M^−1^: the occurrence of 1 : 1 binding under these conditions is confirmed by a Job plot (Fig. 2b and Table S1†). To exclude the possibility that some or all of the guest binding could be with the external hydrophobic surface of Co·H^w^,^13^ at the end of the titration we added an excess of the strongly cavity-binding guest cycloundecanone (CUD: *K* = 10^6^ M^−1^),^14^ at which point the partly-quenched fluorescence of bound luminol was almost completely restored back to its original value (Fig. S2†), implying that it was displaced from the cavity by CUD and the quenching during the titration is due to cavity binding.

**Fig. 3:**
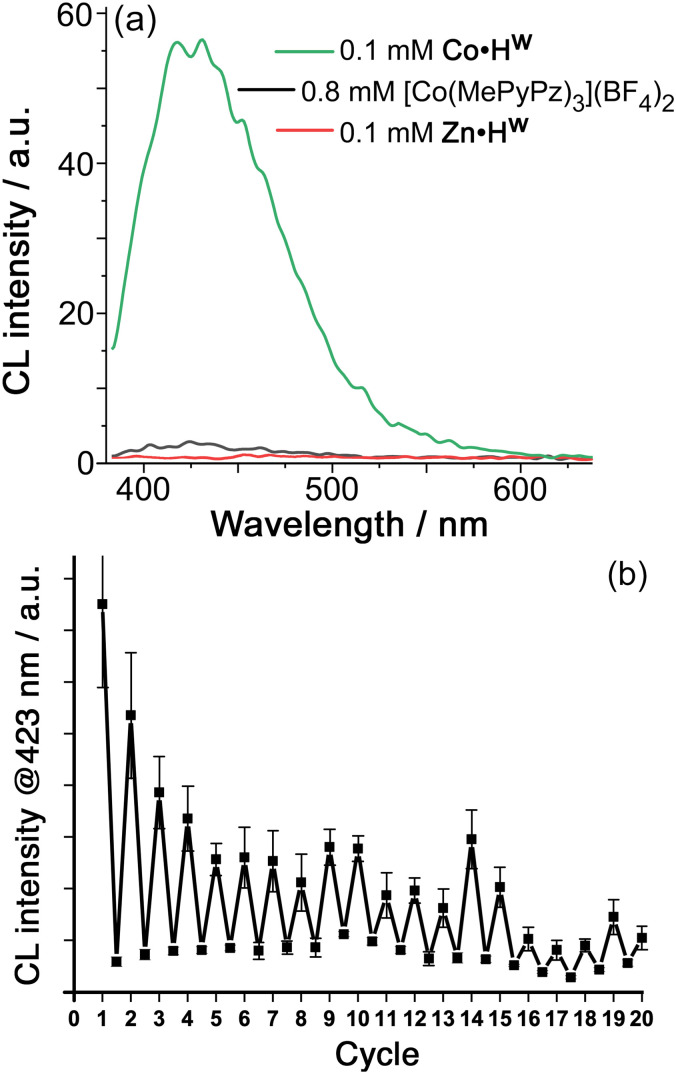
(a) CL spectra generated from luminol (0.1 mM, aqueous borate buffer, pH 8.5) on addition of H_2_O_2_ (3 mM): the control experiments show that CL is only observed when *both* cavity-binding and the presence of Co(ii) ions in the cage apply. (b) Extent of CL generated following repeated addition of fresh portions of luminol (1 equivalent with respect to Co·H^w^) and H_2_O_2_ (30 equivalents) after the CL from the previous cycle had stopped decreasing. Each peak represents the CL intensity immediately after the fresh additions of luminol and H_2_O_2_ to Co·H^w^; the following trough is the CL intensity 30 min later.

We could induce chemiluminescence from the cavity-bound luminol (0.1 mM each of Co·H^w^ and luminol in borate buffer at pH 8.5, meaning that *ca.* 40% of the luminol is cavity-bound) by addition of H_2_O_2_ (Fig. 3, see ESI for experimental details†). The observation of CL implies that the H_2_O_2_ is being activated by a redox reaction with the Co^2+^/Co^3+^ couple of the metal ions in the cage superstructure.^12^ Cyclic voltammetry of Co·H^w^ in aqueous borate buffer (pH 8.5) revealed a wave on the outward sweep at +0.69 V *vs.* Ag/AgCl which we ascribe to the (electrochemically irreversible) Co(ii)/Co(iii) oxidation.^15^ A simple control experiment confirms this: under identical conditions but using the isostructural Zn^2+^ form of the cage Zn·H^w^ we observed no chemiluminescence, which means that the redox activation of H_2_O_2_ requires the Co^2+^/Co^3+^ couple of the cage.^15^

In addition, we can show that the observed chemiluminescence is only associated with that fraction of luminol that is cavity-bound inside Co·H^w^ (≈40%) by replacing 0.1 mM Co·H^w^ by 0.8 mM of the mononuclear complex [Co(MePyPz)_3_]^2+^ – *i.e.* the same number of Co^2+^ ions in an identical coordination environment,^16^‡‡The mononuclear complex [Co(MePyPz)_3_]^2+^ used for the control experiments consists of the expected statistical mixture of 3 : 1 *mer* : *fac* tris-chelate geometries (see ESI† and ref. 16), which is identical to the ratio present in Co·H^w^ (ref. 5–8). but in a form where no guest encapsulation is possible (see ESI for synthesis and characterisation data†). This replacement results in only very weak chemiluminescence from luminol under otherwise identical experimental conditions (Fig. 3). The benefit of cage-based encapsulation is, therefore, clear.

Thus, the observed chemiluminescence in Fig. 3(a) is coming solely from cage-bound luminol which is being oxidised by ROS generated by activation of H_2_O_2_ using the cage-based Co^2+^/Co^3+^ couple. The exact nature of the ROS is not clear due to the complexity of the cascade of reactions that can occur when H_2_O_2_ reacts with a low oxidation-state metal ion: products can include HO˙, HO^−^, HO_2_˙, peroxide and superoxide ions depending on the metal ion and the conditions such as pH.^12^ Necessarily, however, the ROS generated by reaction of H_2_O_2_ with Co·H^w^ will be generated in close proximity to the cage cavity, surrounding the bound luminol, which contributes to the cage-based catalysis of the oxidation reaction in Fig. 1: in particular, any *anionic* ROS generated will tend to accumulate around the cationic cage surface, which is the basis of other cage-catalysed reactions of cavity-bound substrates with surface-bound hydroxide,^13,17^ phenolate,^7^ or peroxymonosulfate anions.^15^ Importantly, whilst metal-catalysed activation of H_2_O_2_ often proceeds *via* inner-sphere mechanisms involving formation of {M–OOH}^*n*+^ intermediates,^12^ this is not always the case and outer-sphere one-electron redox reactions of H_2_O_2_ are known to occur with a range of metal ions when the metal centre is coordinatively saturated, as in Co·H^w^.^15,18^

We note also the crucial role of Co·H^w^ in accumulating hydroxide ions around the cationic surface to generate a high local pH around the bound substrate, which is essential for CL to occur. The intensity of CL from luminol is sensitive to pH for two reasons. Firstly, some of the Co^2+^-catalysed pathways for activation of H_2_O_2_ require base or generate protons.^12^ Secondly, luminol needs to be doubly deprotonated as part of the oxidation process: the two p*K* values in water are 6.7 and 15.1. Whilst the exact mechanism for luminol oxidation is complex and likely to have multiple pathways, this dependence on base concentration is well established^11*a*^ and provides a rationale for the role of Co·H^w^ whose high positive charge results in effective accumulation of hydroxide ions at surface binding sites in the cage faces.^16^ This effect is so strong that the Kemp elimination reaction of benzisoxazole with hydroxide ions is accelerated by >5 orders of magnitude inside the cage cavity of Co·H^w^: even when the *bulk* pD of the reaction solution (in D_2_O) is 8.5, the *local* concentration of DO^−^ ions surrounding the substrate provides an apparent pD of 13.8.^16^

**Fig. 4:**
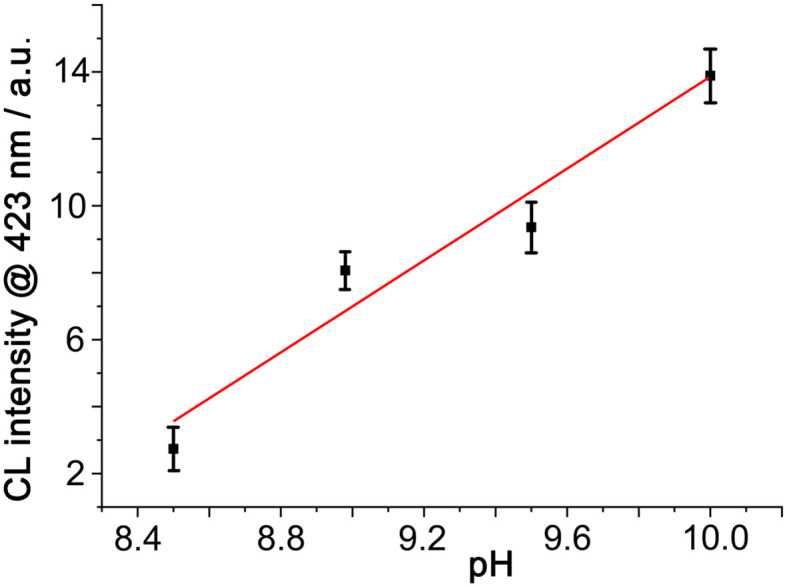
CL intensity from luminol and [Co(MePyPz)_3_](BF_4_)_2_ generated by addition of H_2_O_2_ at different pH values (conditions, and *y*-axis scale, as per Fig. 3 apart from varying pH values).

We can demonstrate that the same anion-accumulation effect occurs here by using a fixed concentration of mononuclear [Co(MePyPz)_3_]^2+^ to activate H_2_O_2_ in the presence of luminol at different pH values (Fig. 4). Under these conditions (i) there is very little CL generated at pH 8.5 (<5% of what was observed in the presence of Co·H^w^, *cf.*Fig. 3a) and (ii) there is an obvious increase in CL intensity with pH above this value. In fact the CL intensity arising from [Co(MePyPz)_3_]^2+^/luminol at pH 10 is about a quarter of what was generated using Co·H^w^ as catalyst at pH 8.5 under otherwise identical conditions: *i.e.* without the effect of Co·H^w^ to accumulate the HO^−^ ions around the cavity-bound luminol, substantially higher pH values are needed to generate even modest CL from luminol.

Overall, this reaction therefore illustrates a rare example of cage-based catalysis in which the metal ions in the cage superstructure act as redox partners in the reaction sequence as well as the cage providing co-location of reaction partners using two orthogonal recognition processes (hydrophobic binding of luminol in the cavity; electrostatic accumulation of anions).^8^ The cage binds the luminol substrate and thereby holds it in close proximity to both (i) the ROS which are the ultimate oxidising agents and are generated at the cage surface surrounding the substrate, and (ii) the hydroxide ions which are essential reaction partners in the oxidation of luminol. The cage also provides, *via* a Co(ii)/Co(iii) couple involving the cage superstructure, the initial redox activation of H_2_O_2_ which makes the reaction possible.^15^ Re-reduction of Co(iii) to Co(ii) to complete a catalytic cycle requires a reducing agent. In aqueous media the strong solvation of hydroxide ions makes them poor reductants,^19^ but it is well known that H_2_O_2_ (present in substantial excess) can be a good reducing agent in basic conditions when it exists as HOO^−^,^20^ with the standard electrode potential for the two-electron O_2_/HOO^−^ couple being +0.08 V.

Addition of further aliquots of luminol and H_2_O_2_ allows the process to be repeated for several cycles; during this time the ^1^H NMR spectrum of signals attributable to Co·H^w^ in the reaction mixture^21^ remain unchanged (Fig. S5†), indicating the stability of catalyst Co·H^w^, though the intensity of CL generated by each luminol/H_2_O_2_ addition does diminish eventually, possibly because accumulation of reaction products inhibits luminol binding (Fig. 3b). Despite this it is clear from Fig. 3b that ≫1 equivalent of luminol is oxidised before the reaction dies, confirming the cage-based catalysis.

**Fig. 5:**
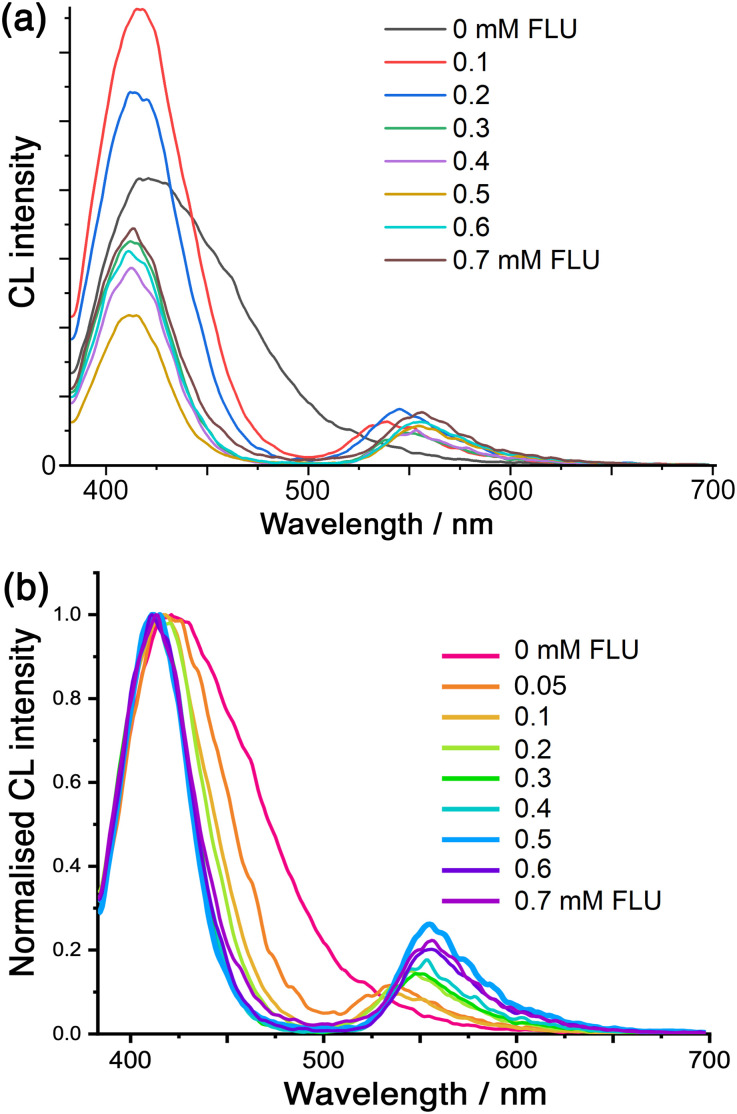
(a) Variation in the CL spectra from luminol (0.1 mM) in the presence of Co·H^w^ (0.1 mM) and H_2_O_2_ at pH 8.5 (conditions as per Fig. 3), following addition of portions of fluorescein (FLU; 0–7 equivalents). (b) Same data as in (a), but drawn with the luminol emission intensity normalised to allow clearer visualisation of the band narrowing associated with re-absorption of the lower-energy CL emission by the FLU absorption around 500 nm (see main text and ref. 22).

Finally, we note that we can use the CL generated by cavity-bound luminol to effect energy-transfer to surface-bound fluorescein (FLU) units. Whilst we have reported examples of *photoinduced* energy- or electron-transfer between chromophores in the cage itself and cavity-bound guests,^9,10^ we now demonstrate energy-transfer from the internal to the external guests across the cage superstructure: the donor (luminol) and acceptor (FLU) components of the pair are brought into proximity by their orthogonal interactions with the cage cavity and surface, respectively.^8^ Accordingly, addition of FLU to a Co·H^w^/luminol mixture (prepared as described earlier; see ESI for experimental details†) shows that, when the CL from luminol is initiated by addition of H_2_O_2_, we see not only progressive quenching of the luminol chemiluminescence, but we also generate sensitised fluorescence at around 550 nm from the surface-bound FLU units (Fig. 5).

Some features of this need detailed comment. Firstly, we see that as more FLU is added the luminol CL band becomes *narrower* (as well as weaker) which is associated with a simple filtering effect: this is, effectively, the ‘trivial’ energy-transfer mechanism whereby that part of the CL emission that overlaps with the FLU absorption maximum is re-absorbed.^22^ Here, that means that the longer-wavelength tail of the luminol CL is absorbed by FLU but the higher-energy CL component is not. The resulting progressive narrowing of the CL band is emphasised in the normalised spectra shown in Fig. 5b. Secondly, we see that there is an initial increase in luminol CL intensity before the expected progressive quenching by FLU. This arises from a sensitisation effect whereby FLU *accelerates* the slow CL decay of luminol, leading to an apparent intensity increase at the early stages of the titration:^22^ this accounts for the rise in CL intensity when the first equivalent of FLU is added to the titration in Fig. 5a.

The sensitised fluorescence from FLU is present between 500–600 nm, and is weak due to partial quenching by the Co(ii) ions in the cage.^6^ Note that no external excitation of FLU is occurring: the key point is that FLU emission is observed only because it is sensitised by the chemiluminescence of luminol.

This energy-transfer is not standard photoinduced energy-transfer as the excited state of the donor is not generated by light absorption: it is an example of CRET (Chemiluminescence Resonance Energy Transfer),^23^ in which the cage (i) binds luminol, (ii) performs redox activation of H_2_O_2_ to initiate the chemiluminescence, and (iii) brings the FLU units into close proximity to generate the cavity-to-surface energy-transfer process. Direct (non-radiative) energy-transfer is likely to occur principally by the Förster mechanism given the singlet excited states of the donor and acceptor species. There is clearly also a contribution from the ‘trivial’ (non-resonance) energy-transfer mechanism, *i.e.* emission of photons from luminol CL followed by re-absorption by fluorescein. This is evident from the change in luminol CL band shape as more FLU is added, with progressive narrowing of the CL band on the low energy side (Fig. 5 and S6†) corresponding to re-absorption of the emitted photons by the increasing concentration of FLU which absorbs strongly in the 500–550 nm region, as reported by others.^22^

Given that the balance between Förster and ‘trivial’ energy-transfer mechanisms will vary during the titration as the balance between surface-bound and free FLU changes, and that the sensitised emission from fluorescein is partly quenched by the Co(ii) ions in the cage,^6^ any quantitative analysis of the energy-transfer efficiency is not possible. The key point is that the effect of Co·H^w^ in bringing together the cavity-bound neutral guest (luminol) as energy-donor, and the surface-bound anionic guests (fluorescein) as energy-acceptors, permits CRET to happen: it is an interesting variation on the cage-to-guest photoinduced energy and electron transfer that we have reported earlier.^9,10^ This ability to trigger energy-transfer with an associated fluorescent response by a chemical signal (addition of H_2_O_2_), rather than by absorption of a photon, is of interest in a range of analytical applications^23^ and is new to coordination cage chemistry.

## Conclusions

A combination of (i) binding luminol as a guest inside a coordination cage host, (ii) redox activation of H_2_O_2_ by the Co(ii) ions in the cage to generate reactive oxygen species around the cage surface, and (iii) accumulation of hydroxide ions (which also participate in the reaction) around the cage surface, result in the oxidation of cage-bound luminol by the ROS and hydroxide ions. This results in chemiluminescence from the caged guest in a manner reminiscent of a lantern. Energy-transfer from this chemiluminescence – by a combination of ‘trivial’ (emission and reabsorption) and Förster non-radiative mechanisms – to surface-bound fluorescein units around the cage exterior generates sensitised fluorescein-based emission by the CRET mechanism. The scope for combining the selectivity of guest binding in the cage cavity, with the cage-based redox activation of H_2_O_2_ to effect oxidation reactions of bound substrates, is broad and represents a substantial new direction in coordination cage based catalysis.

## Conflicts of interest

There are no conflicts to declare.

## Supplementary Material

DT-052-D3DT00689A-s001
