# Supplementary material for: A chemiluminescent lantern: a coordination cage catalysed oxidation of luminol followed by chemiluminescence resonance energy-transfer
Source: Dalton Trans. 2023 Mar 8;52(14):4456–61. doi: 10.1039/d3dt00689a (PMC10071490; doi:10.1039/d3dt00689a)
Supplement: DT-052-D3DT00689A-s001 [file DT-052-D3DT00689A-s001.pdf]

Electronic Supplementary Material (ESI) for Dalton Transactions.
This journal is © The Royal Society of Chemistry 2023

Supporting Information

A chemiluminescent lantern: a coordination cage catalysed oxidation of luminol

Atena B. Solea and Michael D. Ward*

Contents

Experimental data 2
Raw data for determination of the association constant K_a between luminol and Co•H^w 3
Raw data used for Job plot analysis 3
Fluorescence displacement of luminol by cycloundecanone (CU) 4
Characterisation data (NMR, ES-MS) for $[Co(MePyPz)_3](BF_4)_2$ 5
Paramagnetic ^{1}H NMR spectra of Co•H^w before / after a catalytic reaction 6
References 6

Experimental section

General

The cages **M•H^w^** were prepared as their chloride salts as previously described.[1,2] Luminol, H_2O_2 solution 30% and fluorescein were purchased from Avantor, Sigma Aldrich and Acros Organics, respectively, and used without further purification. The H_2O_2 solution was diluted to its final stock concentration with 50 mM borate buffer solution of the appropriate pH for the required experiment. The ligand MePyPz used to make the control complex $[Co(MePyPz)_3](BF_4)_2$ was synthesised as previously reported.[3] Paramagnetic ^1H NMR spectra were recorded in D_2O or CD_3CN on a Bruker AV-300 instrument at 298 K, using the solvent residual peak as internal reference. The chemiluminescence studies were performed on a Cary Eclipse Fluorescence Spectrometer, using the Chemiluminescence mode, with the lamp switched off. The Job plot and fluorescence titrations were carried out at 298 K on a BMG ClarioStar plate reader with 96-well plates.

Chemiluminescence studies

For the chemiluminescence studies, stock solutions of the reactants were prepared as follows: 4 mM luminol in DMSO, 100 mM H_2O_2 in borate buffer (50 mM), 20 mM fluorescein in borate buffer (50 mM), approx. 1 mM **Co•H^w** in 50 mM borate buffer (its exact concentration was determined by UV-Vis, considering an extinction coefficient of 308000 $M^{-1}cm^{-1}$). The chemiluminescent reaction was induced by preparing in a fluorescence cuvette a 1940 µL solution containing 0.1 mM **Co•H^w** (or 0.8 mM Co^{2+} mononuclear complex), 0.1 mM luminol in 50 mM borate buffer at the appropriate pH, into which 60 µL H_2O_2 stock solution (100 mM) was injected. Immediately after injection, the chemiluminescence spectrum was recorded. Similarly, for the CRET studies, in a fluorescence cuvette, to 1940 µL solution containing 0.1 mM **Co•H^w**, 0.1 mM luminol and 0.1-0.7 mM fluorescein, in 50 mM borate buffer, 60 µL H_2O_2 stock solution was injected to induce the chemiluminescent reaction, whose chemiluminescent intensity was immediately recorded.

Fluorescence titrations

For the florescence titration studies, in order to keep the luminol concentration constant, various volumes of a 10 µM stock solution of luminol (2% DMSO in water) were mixed with the corresponding volumes of a **Co•H^w** /luminol 100/10 µM (2% DMSO in water) solution, up to a final volume of 200 µL. The fluorescence spectrum of each well was recorded, using an excitation wavelength of 350 nm. Each titration was performed in triplicate.

Synthesis of $[Co(PyPzMe)_3](BF_4)_2$

In a round bottom flask equipped with a condenser, $Co(BF_4)_2 \bullet 6H_2O$ (16 mg, 0.05 mmol, 1 eq) was refluxed with PyPzMe (48 mg, 0.15 mmol, 3 eq) in 7 mL MeOH for 16 h. The reaction mixture was allowed to reach room temperature and afterwards the solvent was partially removed under reduced pressure. 10 mL Et_2O were added to the solution. The obtained orange precipitate was filtered off and washed with cold H_2O, cold MeOH and cold CH_2Cl_2 and dried under reduced pressure. Obtained: 25 mg of *fac* and *mer* isomers in a 1:3 mixture (4 ligand environments, see main text, ref 15.). Yield: 71 %. ^1H NMR (paramagnetic) (300 MHz, CD_3CN) δ 96.21 (1H, H_{arom}), 93.01 (1H, H_{arom}), 87.13 (1H, H_{arom}), 84.49 (1H, H_{arom}), 73.26 (1H, H_{arom}), 69.59 (1H, H_{arom}), 64.07 (1H, H_{arom}), 62.04 (1H, H_{arom}), 61.17 (1H, H_{arom}), 57.03 (1H, H_{arom}), 55.42 (1H, H_{arom}), 52.67 (1H, H_{arom}), 49.82 (1H, H_{arom}), 47.67 (1H, H_{arom}), 39.17 (1H, H_{arom}), 38.76 (1H, H_{arom}), 17.86 (1H, H_{arom}), 15.30 (1H, H_{arom}), 11.35 (1H, H_{arom}), 9.48 (1H, H_{arom}), -27.84 (3H, CH3), -32.80 (3H, CH3), -33.17 (3H, CH3), -35.88 (3H, CH3). HRMS measured for $[C_{27}H_{27}CoN_9]^{2+}$ ($[Co(PyPzMe)_3]^{2+}$, measured *m/z* 268.0862; calculated *m/z* 268.0855).

Determination of the association constant K_a between luminol and Co•H^w

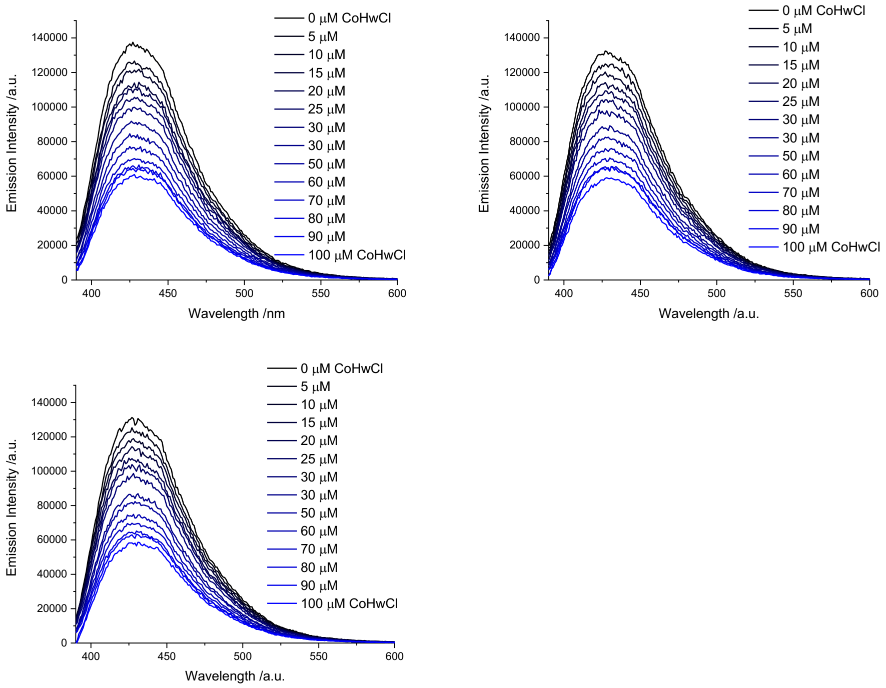

Figure S1. Fluorescence titrations (triplicate) of luminol with Co•H^w. λ_{ex} = 350 nm

Data used for Job plot measurements

Table S1. Determination of the LUM:Co•H^w binding ratio (see main text, Fig. 2b)

x_{LUM}	x_{CoHwCl}	Fluorescence Intensity at 423 nm	Fluorescence quenching / a.u.
0	1	435	435
0.1	0.9	12863	11576.7
0.2	0.8	27321	21856.8
0.3	0.7	45638	31946.6
0.4	0.6	65852	39511.2
0.45	0.55	77373	42555.15
0.5	0.5	86812	43406
0.55	0.45	97222	43749.9
0.6	0.40	108800	43520
0.65	0.35	120130	42045.5
0.7	0.30	129037	38711.1
0.8	0.2	151033	30206.6
0.9	0.1	178724	17872.4
1	0	205057	0

Fluorescence displacement of luminol by cycloundecanone (CU)

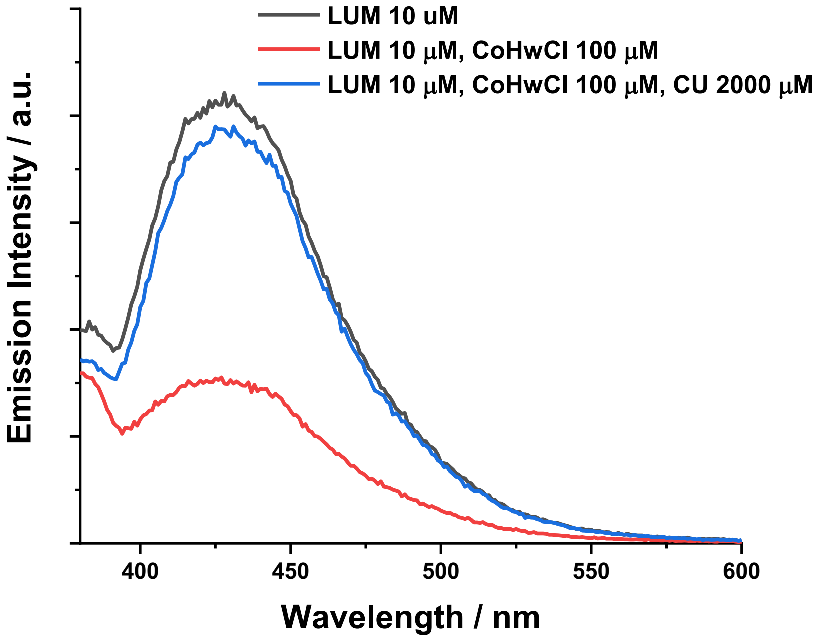

Figure S2. Restoration of the fluorescence signal of luminol upon the addition of excess of cycloundecanone (CU) to the luminol / Co•H^{w} mixture. λ_{ex} = 350 nm

Characterisation of $[Co(MePyPz)_3](BF_4)_2$

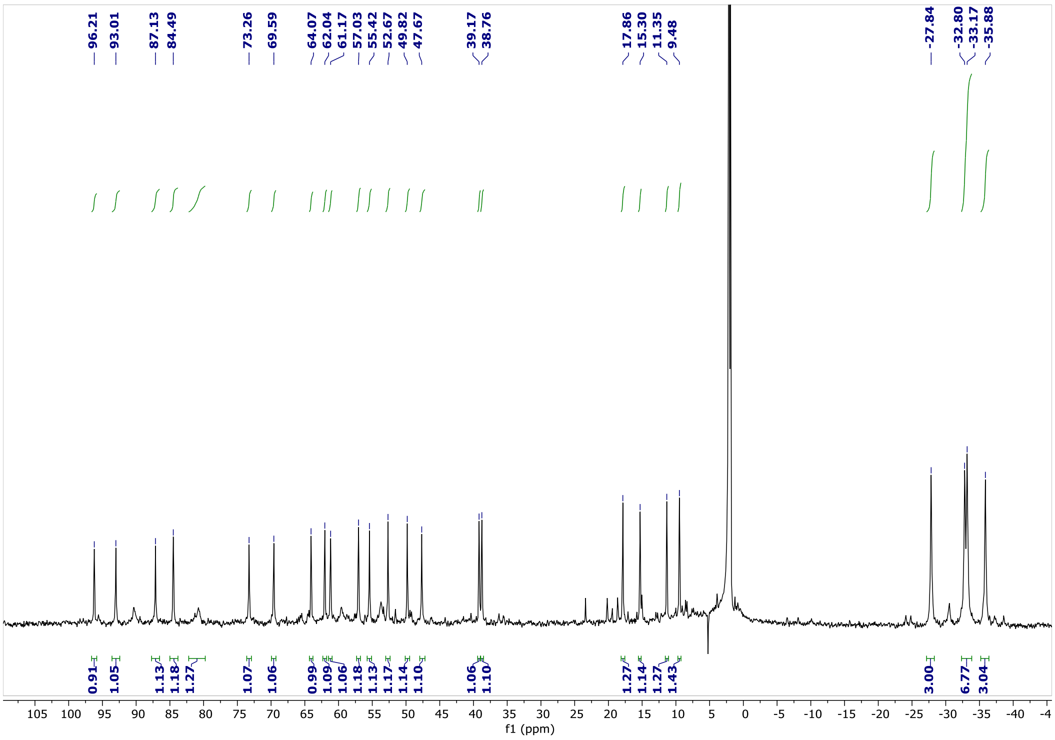

Figure S3. 1H NMR spectrum of $[Co(MePyPz)_3](BF_4)_2$ in CD_3CN, showing the 1:3 mixture of fac and mer isomers, giving four independent ligand environments

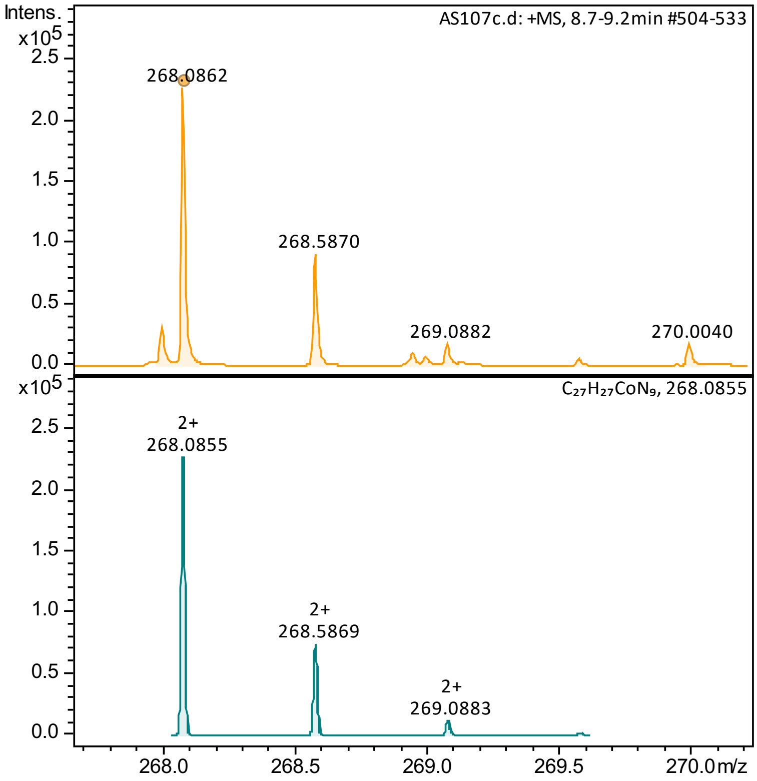

Figure S4. HR-ESMS spectrum of $[Co(MePyPz)_3](BF_4)_2$ in MeCN: measured (top) and simulated (bottom)

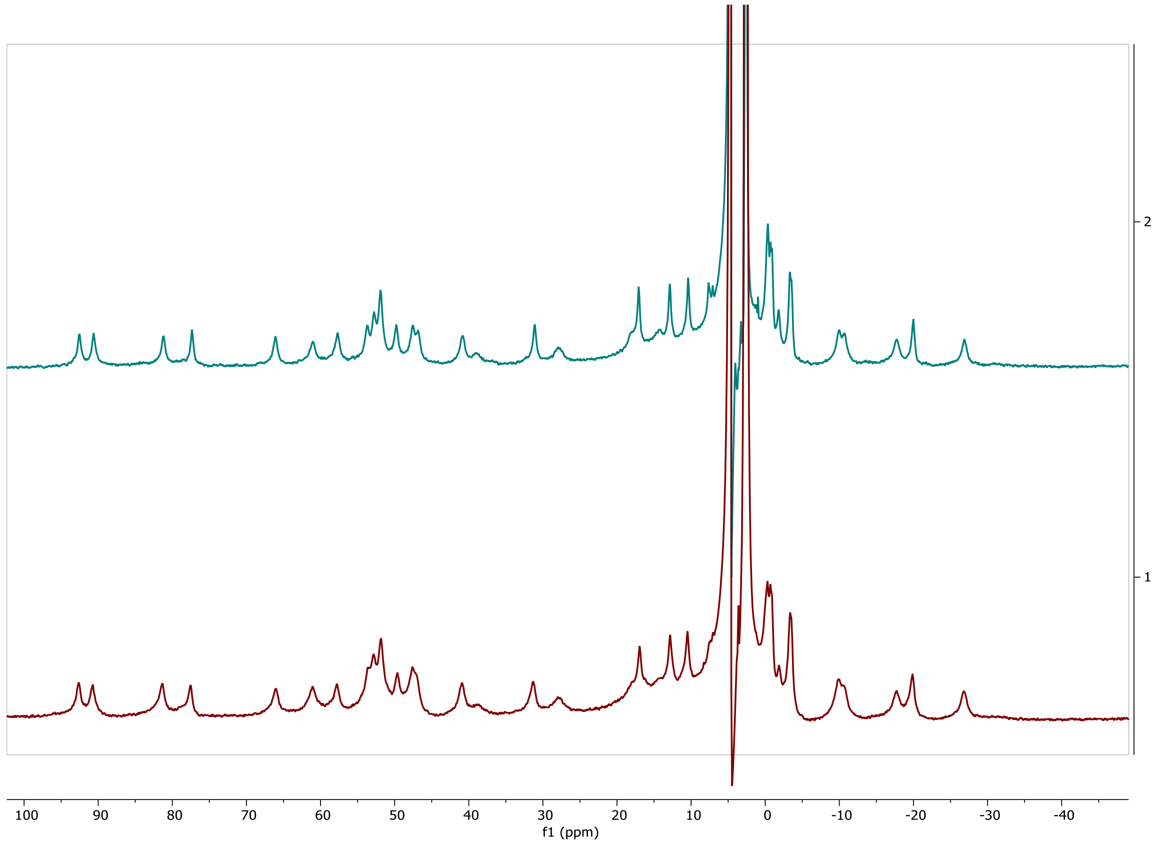

Figure S5. Paramagnetic ^{1}H NMR spectra in D_2O (300 MHz) of the reaction mixture before (top) and after the chemiluminescent reaction (bottom), conditions as in main text Fig. 3, confirming the stability of Co•H^w during a catalytic cycle

References

[1] M. Whitehead, S. Turega, A. Stephenson, C. A. Hunter, and M. D. Ward, *Chem. Sci.,* **2013**, *4*, 2744.

[2] W. Cullen, A. J. Metherell, A. B. Wragg, C. G. P. Taylor, N. H. Williams, and M. D. Ward, *J. Am. Chem. Soc.,* 2018, **140**, 2821.

[3] K. L. Skubi, J. B. Kidd, H. Jung, I. A. Guzei, M. H. Baik, T. P. Yoon, *J. Am. Chem. Soc.* **2017**, *139*, 17186.
